# Utility of Combination Antimicrobial Therapy in Adults with Bloodstream Infections due to Enterobacteriaceae and Non-Fermenting Gram-Negative Bacilli Based on In Vitro Analysis at Two Community Hospitals

**DOI:** 10.3390/antibiotics8010015

**Published:** 2019-02-08

**Authors:** Rachel A. Foster, Casey Troficanto, P. Brandon Bookstaver, Joseph Kohn, Julie Ann Justo, Majdi N. Al-Hasan

**Affiliations:** 1 Department of Pharmacy, Intermountain Healthcare, Murray, UT 84107, USA; rachel.foster@imail.org; 2 Department of Pharmacy, Prisma Health Baptist Hospital, Columbia, SC 29220, USA; casey.troficanto@palmettohealth.org; 3 Department of Clinical Pharmacy and Outcomes Sciences, University of South Carolina College of Pharmacy, Columbia, SC 29208, USA; bookstaver@cop.sc.edu (P.B.B.); justoj@cop.sc.edu (J.A.J.); 4 Department of Pharmacy, Prisma Health Richland Hospital, Columbia, SC 29203, USA; joseph.kohn@palmettohealth.org; 5 University of South Carolina School of Medicine, Columbia, SC 29209, USA; 6 Department of Medicine, Division of Infectious Diseases, Palmetto Health University of South Carolina Medical Group, Columbia, SC 29203, USA

**Keywords:** bacteremia, antibiotics, *Escherichia coli*, *Klebsiella* species, *Pseudomonas aeruginosa*, extended-spectrum beta-lactamases, AmpC, sepsis

## Abstract

This study examined the utility of combination therapy for bloodstream isolates of *Enterobacteriaceae* and non-fermenting Gram-negative bacilli (NFGN) from adults at two community hospitals from January 2010 through to June 2015. Changes to in vitro antimicrobial susceptibilities by adding ciprofloxacin or gentamicin to third-generation cephalosporins (3GC) were examined overall and in patients with risk factors for 3GC resistance. Overall ceftriaxone susceptibility among *Enterobacteriaceae* was 996/1063 (94%) and 247/295 (84%) in patients with 3GC resistance risk factors. Susceptibilities increased marginally by adding ciprofloxacin or gentamicin (mean difference 2.4% (95% CI 1.5, 3.4) and 3.0% (95% CI 2.0, 4.0), respectively, overall and 5.4% (95% CI 2.8, 8.0) and 7.1% (95% CI 4.2, 10.1), respectively, in patients with risk factors). Eighty-three of 105 (79%) NFGN were susceptible to ceftazidime overall and 20/29 (69%) in patients with prior beta-lactam use. Overall mean increase in susceptibilities was 15.2% (95% CI: 8.3, 22.2) and 17.1% (95% CI: 9.8, 24.5) for ciprofloxacin and gentamicin combinations, respectively; and 27.6% (95% CI: 10.3, 44.9) for either one with recent beta-lactam use. In this setting, empirical combination therapy had limited utility for *Enterobacteriaceae* bloodstream isolates but provided significant additional antimicrobial coverage to ceftazidime for NFGN, particularly in patients with prior beta-lactam use.

## 1. Introduction

Increasing antimicrobial resistance rates continue to challenge the approach to empirical antimicrobial selection [1,2]. The use of two antimicrobial agents from different classes may increase the appropriateness of empirical coverage [3]. However, the role of empirical combination antimicrobial therapy in Gram-negative bloodstream infections (BSI) remains uncertain. Earlier studies demonstrated inconsistent conclusions [4,5,6,7,8,9,10,11]. The results of more recent studies suggest no benefit from the nonstratified use of combination therapy in Gram-negative BSI [12,13,14,15,16,17,18]. However, antimicrobial resistance rates of Gram-negative bacilli to broad-spectrum beta-lactams, such as third-generation cephalosporins (3GC), remain high in the setting of prior beta-lactam use among other risk factors for 3GC resistance [19,20,21]. It is hypothesized that the addition of a fluoroquinolone or an aminoglycoside to 3GC may increase the appropriateness of empirical antimicrobial regimens in these high-risk patients. The use of combination therapy in these settings may be an alternative antimicrobial stewardship strategy to the empirical use of carbapenems or new, more expensive beta-lactam/extended-spectrum beta-lactamase (ESBL) inhibitors. This study examined the potential benefit of combination therapy on the appropriateness of empirical antimicrobial regimens in adult patients with BSI due to *Enterobacteriaceae* and non-fermenting Gram-negative bacilli (NFGN) overall and after stratification based on risk factors for 3GC resistance at initial presentation.

## 2. Results

### 2.1. Clinical Characteristics and Microbiology

A total of 1168 patients with BSI were included in the study; 1063 (91%) due to *Enterobacteriaceae* and 105 (9%) due to NFGN. *Escherichia coli* (597; 51%) was the most common bloodstream isolate overall, followed by *Klebsiella pneumoniae* (225; 19%), *Proteus mirabilis* (78; 7%), *Enterobacter* spp. (76; 6%), and other *Enterobacteriaceae* (87; 8%). *Pseudomonas aeruginosa* (70; 6%) was the most common among NFGN, followed by *Acinetobacter* spp. (17; 1.5%) and other NFGN (18; 1.5%). Demographics and clinical characteristics of patients with BSI due to *Enterobacteriaceae* and NFGN are shown in Table 1.

### 2.2. Additional Antimicrobial Coverage of Combination Regimens 

Among *Enterobacteriaceae* bloodstream isolates, 94% were susceptible to ceftriaxone with a marginal change in susceptibilities by adding either ciprofloxacin or gentamicin (Table 2). In patients with risk factors for 3GC resistance, 84% of *Enterobacteriaceae* bloodstream isolates were susceptible to ceftriaxone. Adding ciprofloxacin or gentamicin to ceftriaxone improved susceptibilities of combination regimen by only 5.4% and 7.1%, respectively.

A substantial change in susceptibility was observed with the addition of either ciprofloxacin or gentamicin to ceftazidime among NFGN (mean difference 15.2% and 17.1%, respectively). Antimicrobial susceptibility of NFGN to ceftazidime was 69% in patients with prior use of beta-lactams within the past 30 days. The addition of ciprofloxacin or gentamicin to ceftazidime increased susceptibility rates to 97% for each respective combination regimen. Similar patterns were observed among bloodstream isolates of *P. aeruginosa*, where ceftazidime susceptibility rates were 81% and 96% in the presence or absence of recent beta-lactam exposure, respectively. The addition of either ciprofloxacin or gentamicin significantly improved the appropriateness of the empirical regimen only in patients with prior beta-lactam use (Table 2).

### 2.3. Correlation between Susceptibilities of Different Antimicrobial Classes

Overall, 83% and 93% of *Enterobacteriaceae* bloodstream isolates were susceptible to ciprofloxacin and gentamicin, respectively. When stratified by susceptibility to ceftriaxone, only 39% and 48% of ceftriaxone-nonsusceptible *Enterobacteriaceae* were susceptible to ciprofloxacin and gentamicin, respectively (Table 3). Susceptibilities for these agents among ESBL-producing *Enterobacteriaceae* were even lower (21% and 31%, respectively), limiting the utility of combination therapy when needed the most. There was a positive correlation between antimicrobial susceptibilities of ceftriaxone and both ciprofloxacin and gentamicin in *Enterobacteriaceae* bloodstream isolates (Table 4). This correlation was mostly driven by bloodstream isolates of *E. coli*, *Klebsiella* spp., and *Proteus mirabilis*. There was a lack of correlation between susceptibilities to ceftriaxone and either combination agent among *Enterobacter*, *Serratia*, and *Citrobacter* spp. bloodstream isolates. 

Overall antimicrobial susceptibility rates to ciprofloxacin and gentamicin among NFGN were 85% and 88%, respectively. Susceptibility to ciprofloxacin and gentamicin was retained among 73% and 82% of ceftazidime-nonsusceptible NFGN, respectively (Table 3). No correlation between antimicrobial susceptibilities of ceftazidime and either ciprofloxacin or gentamicin was detected in NFGN (Table 4).

## 3. Discussion

### 3.1. Clinical Applications of Study Findings 

This study demonstrates that the empirical use of combination antimicrobial therapy has limited utility in patients with BSI due to *Enterobacteriaceae*. Compared to monotherapy with ceftriaxone, the addition of ciprofloxacin or gentamicin provided only marginal difference in the susceptibilities of *Enterobacteriaceae* bloodstream isolates overall. Even in patients with specific risk factors for antimicrobial resistance where susceptibility of *Enterobacteriaceae* bloodstream isolates to ceftriaxone was only 84%, combination therapy with ciprofloxacin or gentamicin did not provide considerable gains (89% and 91%, respectively). These susceptibility rates of combination regimens are unlikely to meet healthcare providers’ expectations for empirical antimicrobial regimens in patients with potentially life-threatening infections, such as BSI [22,23,24]. Moreover, considering the potential adverse events from either ciprofloxacin or gentamicin and high numbers needed to treat to improve the appropriateness of empirical therapy by adding either agent to ceftriaxone (19 and 14, based on mean differences of 5.4% and 7.1%, respectively), it is likely the potential risks of combination therapy exceed the benefits in this setting. The current results do not support the use of combination antimicrobial therapy as a carbapenem-sparing option in patients with risk factors for BSI due to ESBL-producing *Enterobacteriaceae* in our local population or others with similar antimicrobial resistance patterns. 

Conversely, in patients with BSI due to NFGN, including *P. aeruginosa*, combination therapy significantly improved the appropriateness of the empirical regimen over ceftazidime monotherapy, particularly in the setting of recent beta-lactam use. This emphasizes the concept of stratifying patients based on prior antimicrobial use in order to improve the selection of empirical antimicrobial therapy. Applying this stratified empirical approach, the use of combination therapy would have been entertained in only 30% (21/70) of patients with *P. aeruginosa* BSI. Among this subset of patients with *P. aeruginosa* BSI and prior beta-lactam exposure, the numbers needed to treat to improve appropriateness of empirical therapy was only five (based on a mean difference of 19%), making this a reasonable strategy in critically-ill patients. For the remaining patients without prior beta-lactam use, ceftazidime monotherapy provided appropriate coverage for 96% of *P. aeruginosa* bloodstream isolates, negating the need for a combination agent. The small number and heterogeneity of other NFGN in this study make it difficult to draw meaningful conclusions regarding this group of bacteria. Further studies to delineate the potential benefits of combination antimicrobial regimens in these patients are warranted. 

### 3.2. Potential Explanations for Correlation between Susceptibilities of Different Antimicrobial Classes

The discordant results regarding the utility of combination regimens in *Enterobacteriaceae* and *P. aeruginosa* bloodstream isolates may be explained by the different resistance mechanisms present in the respective bacteria. The study demonstrates a fair agreement between susceptibilities of ceftriaxone and ciprofloxacin, and a moderate agreement between ceftriaxone and gentamicin susceptibilities among *Enterobacteriaceae* bloodstream isolates (Table 4). This is conceivable given that phenotypic screening test for ESBL production was positive in the majority of ceftriaxone-nonsusceptible *Enterobacteriaceae* in this study (42/66; 64%). It is likely these isolates often carry resistance genes to other antimicrobial classes such as fluoroquinolones or aminoglycosides on the same or other plasmids [25]. Although *P. aeruginosa* can carry ESBL on plasmids, this remains relatively uncommon compared to *Enterobacteriaceae* [25,26,27]. More common resistance mechanisms to ceftazidime among *P. aeruginosa* isolates include chromosomally-mediated AmpC-production, efflux pumps, and outer membrane protein alterations or mutations [26,27]. However, multidrug efflux pumps may contribute to resistances to ceftazidime and other classes of antimicrobials in *P. aeruginosa* [28].

### 3.3. Impact of Antimicrobial Utilization in Hospitals on Choice of Combination Agent

It was noted in this study that fluoroquinolone and aminoglycoside combinations provided comparable antimicrobial susceptibility results for NFGN, including *P. aeruginosa*. This is likely explained by the overall low utilization of fluoroquinolones in the two hospitals included in this study (mean of 30 days of therapy per 1000 patient-days during the study period). This has contributed to overall low resistance rates to fluoroquinolones in NFGN, which are predominantly hospital-onset bacteria. However, in institutions with relatively higher fluoroquinolone utilization and fluoroquinolone resistance rates among *P. aeruginosa* and other NFGN, it is expected that aminoglycoside combinations would be more beneficial than fluoroquinolone combination regimens. Knowledge of local hospital epidemiology, antimicrobial utilization, and antimicrobial resistance rates is essential to determine the potential benefits of either combination regimen in each institution.

### 3.4. Antimicrobial Stewardship Implications

The results of this study offer insights into the utility of combination antibiograms as tools for informed antimicrobial prescribing in hospitals [29]. However, we caution antimicrobial stewardship programs to selectively report combination antibiograms for bacteria only in settings where it would be clinically useful in order to minimize misuse and misinterpretation. The results of the current study argue that reporting a combination antibiogram in similar hospitals is only useful for *P. aeruginosa* and possibly other NFGN in the setting of recent beta-lactam use. It would also be useful for stewardship teams to list risk factors for BSI due to *P. aeruginosa*, such as immune compromised hosts and prolonged hospitalization in the footnotes of such combination antibiograms [30]. 

### 3.5. Strengths and Limitations

The study used a unique approach to identify patients with Gram-negative BSI who may benefit from empirical combination antimicrobial therapy. It examined the utility of combination regimens based on predicted risk of antimicrobial resistance at initial presentation, and then confirmed these findings based on correlation between actual susceptibilities of bloodstream isolates to different antimicrobial classes. 

Limitations of this study include enrollment of patients with BSI from two hospitals from a single healthcare system subject to local epidemiology, antimicrobial resistance, and specific patient populations. The study results may not be generalizable to other institutions with different hospital epidemiology and antimicrobial resistance patterns. The study did not examine antimicrobial susceptibility rates of amikacin since it was not consistently performed in bloodstream isolates throughout the study period. This may have underestimated the potential benefits from aminoglycoside combinations in *Enterobacteriaceae* [31]. Moreover, the current investigation did not examine susceptibilities to the more recent, novel beta-lactam/beta-lactamase inhibitors, such as ceftolozane–tazobactam and ceftazidime–avibactam, since these agents were not available at the time of study. It is conceivable that in vitro antimicrobial susceptibility rates to these agents may be as high as combination regimens, particularly for *P. aeruginosa* [32,33]. However, the global use of these new agents is concerning given the excessive cost and potential induction of antimicrobial resistance once used in a large scale. Use of combination empirical therapy may provide additional options to spare the use of these new agents and augment ongoing antimicrobial stewardship efforts. In addition, the current study was focused on examination of in vitro antimicrobial susceptibility testing of bloodstream isolates. Potential synergy of combination regimens and clinical outcomes were not assessed. Future, preferably multicenter, clinical studies examining the effectiveness of empirical combination antimicrobial therapy in patients with BSI due to *P. aeruginosa* in the setting of recent beta-lactam use would yield highly valuable results. Finally, molecular and phylogenic testing was not performed to identify specific resistance mechanisms of bloodstream isolates and determine *E. coli* group, respectively.

## 4. Materials and Methods

### 4.1. Setting

The study was conducted at Palmetto Health Richland (multidisciplinary community teaching hospital) and Palmetto Health Baptist (multidisciplinary community hospital) in Columbia, South Carolina, USA. Both hospitals combine for over 1000 licensed beds. The Palmetto Health institutional Review Board approved the study and waived informed consent.

### 4.2. Study Design and Definitions

Hospitalized adults with first episodes of monomicrobial BSI due to aerobic Gram-negative bacilli from 1 January 2010 to 30 June 2015 at Palmetto Health Hospitals in Columbia, SC, USA were identified through clinical decision support software and microbiology laboratory reports (*n* = 1168). Receipt of prior antimicrobials was determined from medication administration records and clinical notes from current or prior visits, electronic prescriptions in medical records from prior visits to affiliated hospitals or ambulatory clinics, and third-party pharmacy adjudication claims available in the electronic medical records. In vitro antimicrobial susceptibilities were determined by the VITEK^®^ 2 system using the Clinical and Laboratory Standards Institute (CLSI) criteria. The antimicrobial drugs included on the susceptibility panel were amoxicillin/clavulanate, ampicillin, piperacillin/tazobactam, cefazolin, cefoxitin, ceftriaxone, ceftazidime, cefepime, ertapenem, meropenem, gentamicin, tobramycin, ciprofloxacin, levofloxacin, and sulfamethoxazole/trimethoprim. Screening for ESBL production by the disk diffusion method using cefotaxime/clavulanate combination disks was performed in *Enterobacteriaceae* bloodstream isolates that were nonsusceptible in vitro to any 3GC. Risk factors for 3GC resistance among patients with *Enterobacteriaceae* BSI included prior beta-lactam or fluoroquinolone use within the past 3 months, prior infections or colonization with ESBL-producing *Enterobacteriaceae* within the past 12 months, and recent outpatient gastrointestinal or genitourinary procedures within the past one month as previously described [20]. Receipt of beta-lactams within the past one month was considered a risk factor for 3GC resistance in patients with BSI due to NFGN [21]. Antimicrobial susceptibilities to a 3GC, a fluoroquinolone (ciprofloxacin), and an aminoglycoside (gentamicin) were recorded. The 3GC was defined as ceftriaxone for *Enterobacteriaceae* and ceftazidime for NFGN. The bloodstream isolate was considered susceptible to a combination regimen if it was in vitro susceptible to either or both agents in that combination.

### 4.3. Statistical Analysis

Matched pairs mean difference with 95% confidence intervals (CI) was calculated to examine statistical significance of a change in antimicrobial susceptibility with the addition of a combination agent (ciprofloxacin or gentamicin) to a 3GC in bloodstream isolates of *Enterobacteriaceae* and NFGN. A stratified analysis was performed based on the presence of risk factors for 3GC resistance at initial presentation. The utility of combination therapy for BSI due to *Enterobacteriaceae* and NFGN was examined in patients with and without risk factors for 3GC resistance. 

Kappa coefficients with 95% CI were calculated to assess correlation in susceptibilities between the 3GC and the respective combination agent (ciprofloxacin or gentamicin) for *Enterobacteriaceae*, NFGN and subsets of bacteria from each group. For simplicity of statistical analysis, bloodstream isolates were classified as either susceptible or nonsusceptible based on in vitro antimicrobial susceptibility testing results using CLSI criteria.

The level of significance for statistical testing was defined as *p* < 0.05. JMP Pro (version 12.0; SAS Institute, Cary, NC, USA) was used for all statistical analysis.

## 5. Conclusions

Empirical combination antimicrobial therapy had limited utility in *Enterobacteriaceae* BSI in this patient population, even in the presence of risk factors for 3GC resistance. Conversely, combination regimens provided significant additional in vitro antimicrobial coverage to ceftazidime in NFGN. Patients with BSI due to *P. aeruginosa* and other NFGN, particularly those who used beta-lactams within the past 30 days, benefited the most from combination therapy. Identification of patients with BSI due to *P. aeruginosa* using clinical risk factors and/or rapid diagnostics and stratification by recent beta-lactam use may be considered in the decision to use empirical combination therapy. 

## Figures and Tables

**Table 1:** Demographics and clinical characteristics of patients with bloodstream infections.

Variable	*Enterobacteriaceae* (*n* = 1063)	NFGN (*n* = 105)
Age in y, median (IQR)	65 (54-77)	63 (51-75)
Female sex	575 (54)	45 (43)
Ethnicity		
White	497 (47)	46 (44)
African American	529 (50)	54 (51)
Other	37 (3)	5 (5)
Diabetes mellitus	415 (39)	36 (34)
End-stage renal disease	106 (10)	14 (13)
Liver cirrhosis	51 (5)	4 (4)
Cancer	175 (16)	26 (25)
Immune compromised host	118 (8)	25 (24)
Indwelling central venous catheter	207 (19)	34 (32)
Indwelling urinary catheterization	123 (12)	14 (13)
Residence at skilled nursing facility	164 (15)	10 (10)
Recent hospitalization^*^	328 (31)	42 (40)
Hospital-acquired infection	228 (21)	38 (36)
Pitt bacteremia score ≥4	233 (22)	24 (23)

Data are shown as number (%) unless otherwise specified. NFGN: Non-fermenting Gram-negative bacilli; IQR: Interquartile range. * Within 90 days of bloodstream infection.

**Table 2:** Antimicrobial susceptibility of bloodstream isolates in patients with and without risk factors for third-generation cephalosporin resistance.

Bacteria	3GC n (%)	3GC/FQ n (%)	MD (95% CI)	*p*	3GC/AG n (%)	MD (95% CI)	*p*
*Enterobacteriaceae*							
Overall (*n* = 1063)	996 (94)	1022 (96)	2.4 (1.5, 3.4)	<0.001	1028 (97)	3.0 (2.0, 4.0)	<0.001
No 3GC-R risk factors* (*n* = 768)	750 (98)	760 (99)	1.3 (0.5, 2.1)	0.002	761 (99)	1.4 (0.6, 2.3)	<0.001
3GC-R risk factors* (*n* = 295)	247 (84)	263 (89)	5.4 (2.8, 8.0)	<0.001	268 (91)	7.1 (4.2, 10.1)	<0.001
*Non-fermenting Gram-negative bacilli*							
Overall (*n* = 105)	83 (79)	99 (94)	15.2 (8.3, 22.2)	<0.001	101 (96)	17.1 (9.8, 24.5)	<0.001
No recent use of beta-lactams+ (*n* = 76)	63 (83)	71 (93)	10.5 (3.7, 17.6)	0.004	73 (96)	13.2 (5.4, 20.9)	0.001
Recent beta-lactam use+ (*n* = 29)	20 (69)	28 (97)	27.6 (10.3, 44.9)	0.003	28 (97)	27.6 (10.3, 44.9)	0.003
*Pseudomonas aeruginosa*							
Overall (*n* = 70)	64 (91)	70 (100)	8.6 (1.8, 15.3)	0.01	70 (100)	8.6 (1.8, 15.3)	0.01
No recent use of beta-lactams+ (*n* = 49)	47 (96)	49 (100)	4.1 (-1.7, 9.8)	0.16	49 (100)	4.1 (-1.7, 9.8)	0.16
Recent beta-lactam use+ (*n* = 21)	17 (81)	21 (100)	19.0 (7.1, 37.4)	0.04	21 (100)	19.0 (7.1, 37.4)	0.04

3GC: Third-generation cephalosporin (ceftriaxone for *Enterobacteriaceae* and ceftazidime for non-fermenters); FQ: Fluoroquinolone (ciprofloxacin); MD: Mean difference; CI: Confidence interval; AG: Aminoglycoside (gentamicin); 3GC-R: Third-generation cephalosporin resistance. * Risk factors for third-generation cephalosporin resistance include prior beta-lactam or fluoroquinolone use within the past 3 months, prior infections or colonization with extended-spectrum beta-lactamase producing *Enterobacteriaceae* within the past 12 months, and recent outpatient gastrointestinal or genitourinary procedures within the past one month [20]. + Within 30 days of bloodstream infection.

**Table 3:** Antimicrobial susceptibilities of bloodstream isolates to combination agent based on susceptibility to third-generation cephalosporins.

Bacteria	Ciprofloxacin	*p*	Gentamicin	*p*
*Enterobacteriaceae*	880/1058 (83)		997/1053 (93)	
Ceftriaxone-susceptible Ceftriaxone-nonsusceptible	854/992 (86) 26/66 (39)	<0.001	945/987 (96) 32/66 (48)	<0.001
NFGN	88/104 (85)		92/105 (88)	
Ceftazidime-susceptible Ceftazidime-nonsusceptible	72/82 (88) 16/22 (73)	0.08	74/83 (89) 18/22 (82)	0.35

Data are shown as number of susceptible isolates/number of isolates tested (percentage of susceptible isolates). NFGN: Non-fermenting Gram-negative bacilli.

**Table 4:** Correlation between in vitro antimicrobial susceptibilities of bloodstream isolates to third-generation cephalosporins and combination agents.

Bacteria	Ciprofloxacin		Gentamicin	
	κ (95% CI)	*p*	κ (95% CI)	*p*
*Enterobacteriaceae*	0.27 (0.19, 0.34)	<0.001	0.45 (0.35, 0.56)	<0.001
*E. coli*, *Klebsiella* spp. and *P. mirabilis*	0.30 (0.22, 0.38)	<0.001	0.52 (0.41, 0.63)	<0.001
*Enterobacter*, *Serratia* and *Citrobacter* spp.	-0.07 (-0.10, 0.04)	0.38	-0.06 (-0.09, 0.03)	0.47
Non-fermenting Gram-negative bacilli	0.17 (-0.05, 0.38)	0.08	0.09 (-0.12, 0.29)	0.35
*P. aeruginosa*	-0.10 (-0.16, 0.05)	0.39	-0.04 (-0.09, 0.01)	0.66
Other non-fermenters	0.21 (-0.09, 0.51)	0.17	-0.12 (-0.43, 0.19)	0.45

κ: Kappa coefficient; CI: confidence intervals.

## References

[1] Boucher H.W., Talbot G.H., Bradley J.S., Edwards J.E., Gilbert D., Rice L.B., Scheld M., Spellberg B., Bartlett J. (2009). Bad bugs, no drugs: no ESKAPE! An update from the Infectious Diseases Society of America. Clin. Infect. Dis..

[2] Decker B., Masur H. (2015). Bad bugs, no drugs: are we part of the problem, or leaders in developing solutions?. Crit. Care Med..

[3] Martínez J.A., Cobos-Trigueros N., Soriano A., Almela M., Ortega M., Marco F., Pitart C., Sterzik H., Lopez J., Mensa J. (2010). Influence of empiric therapy with a beta-lactam alone or combined with an aminoglycoside on prognosis of bacteremia due to gram-negative microorganisms. Antimicrob. Agents Chemother..

[4] Leibovici L., Paul M., Poznanski O., Drucker M., Samra Z., Konigsberger H., Pitlik S.D. (1997). Monotherapy versus beta-lactam-aminoglycoside combination treatment for gram-negative bacteremia: a prospective, observational study. Antimicrob. Agents Chemother..

[5] Korvick J.A., Bryan C.S., Farber B., Beam T.R., Schenfeld L., Muder R.R., Weinbaum D., Lumish R., Gerding D.N., Wagener M.M. (1992). Prospective observational study of *Klebsiella* bacteremia in 230 patients: outcome for antibiotic combinations versus monotherapy. Antimicrob. Agents Chemother..

[6] Hilf M., Yu V.L., Sharp J., Zuravleff J.J., Korvick J.A., Muder R.R. (1989). Antibiotic therapy for *Pseudomonas aeruginosa* bacteremia: outcome correlations in a prospective study of 200 patients. Am. J. Med..

[7] Chamot E., Boffi El Amari E., Rohner P., Van Delden C. (2003). Effectiveness of combination antimicrobial therapy for *Pseudomonas aeruginosa* bacteremia. Antimicrob. Agents Chemother..

[8] Chow J.W., Yu V.L. (1999). Combination antibiotic therapy versus monotherapy for gram-negative bacteraemia: a commentary. Int. J. Antimicrob. Agents.

[9] Klibanov O.M., Raasch R.H., Rublein J.C. (2004). Single versus combined antibiotic therapy for gram-negative infections. Ann. Pharmacother..

[10] Safdar N., Handelsman J., Maki D.G. (2004). Does combination antimicrobial therapy reduce mortality in Gram-negative bacteraemia? A meta-analysis. Lancet Infect. Dis..

[11] Al-Hasan M.N., Wilson J.W., Lahr B.D., Thomsen K.M., Eckel-Passow J.E., Vetter E.A., Tleyjeh I.M., Baddour L.M. (2009). Beta-lactam and fluoroquinolone combination antibiotic therapy in bacteremia caused by gram-negative bacilli. Antimicrob. Agents. Chemother..

[12] Vardakas K.Z., Tansarli G.S., Bliziotis I.A., Falagas M.E. (2013). β-Lactam plus aminoglycoside or fluoroquinolone combination versus β-lactam monotherapy for *Pseudomonas aeruginosa* infections: a meta-analysis. Int. J. Antimicrob. Agents.

[13] Peña C., Suarez C., Ocampo-Sosa A., Murillas J., Almirante B., Pomar V., Aguilar M., Granados A., Calbo E., Rodríguez-Baño J. (2013). Spanish Network for Research in Infectious Diseases (REIPI). Effect of adequate single-drug vs combination antimicrobial therapy on mortality in *Pseudomonas aeruginosa* bloodstream infections: a post hoc analysis of a prospective cohort. Clin. Infect. Dis..

[14] Kim Y.J., Jun Y.H., Kim Y.R., Park K.G., Park Y.J., Kang J.Y., Kim S.I. (2014). Risk factors for mortality in patients with *Pseudomonas aeruginosa* bacteremia; retrospective study of impact of combination antimicrobial therapy. BMC Infect. Dis..

[15] Paul M., Lador A., Grozinsky-Glasberg S., Leibovici L. (2014). Beta lactam antibiotic monotherapy versus beta lactam-aminoglycoside antibiotic combination therapy for sepsis. Cochrane. Database Syst. Rev..

[16] Yoon Y.K., Kim H.A., Ryu S.Y., Lee E.J., Lee M.S., Kim J., Park S.Y., Yang K.S., Kim S.W. (2017). Tree-structured survival analysis of patients with *Pseudomonas aeruginosa* bacteremia: A multicenter observational cohort study. Diagn. Microbiol. Infect. Dis..

[17] Ong D.S.Y., Frencken J.F., Klein Klouwenberg P.M.C., Juffermans N., van der Poll T., Bonten M.J.M., Cremer O.L., MARS consortium (2017). Short-course adjunctive gentamicin as empirical therapy in patients with severe sepsis and septic shock: a prospective observational cohort study. Clin. Infect. Dis..

[18] Justo J.A., Bookstaver P.B., Kohn J., Albrecht H., Al-Hasan M.N. (2018). Combination versus monotherapy for gram-negative bloodstream infections: matching by predicted prognosis. Int. J. Antimicrob. Agents.

[19] Tumbarello M., Trecarichi E.M., Bassetti M., De Rosa F.G., Spanu T., Di Meco E., Losito A.R., Parisini A., Pagani N., Cauda R. (2011). Identifying patients harboring extended-spectrum-beta-lactamase-producing *Enterobacteriaceae* on hospital admission: derivation and validation of a scoring system. Antimicrob. Agents Chemother..

[20] Augustine M.R., Testerman T.L., Justo J.A., Bookstaver P.B., Kohn J., Albrecht H., Al-Hasan M.N. (2017). Clinical risk score for prediction of extended-spectrum beta-lactamase producing *Enterobacteriaceae* in bloodstream isolates. Infect. Control Hosp. Epidemiol..

[21] Al-Jaghbeer M.J., Justo J.A., Owens W., Kohn J., Bookstaver P.B., Hucks J., Al-Hasan M.N. (2018). Risk factors for pneumonia due to beta-lactam-susceptible and beta-lactam-resistant *Pseudomonas aeruginosa*: a case-case-control study. Infection.

[22] Kalil A.C., Metersky M.L., Klompas M., Muscedere J., Sweeney D.A., Palmer L.B., Napolitano L.M., O’Grady N.P., Bartlett J.G., Carratalà J. (2016). Management of adults with hospital-acquired and ventilator-associated pneumonia: 2016 Clinical Practice Guidelines by the Infectious Diseases Society of America and the American Thoracic Society. Clin. Infect. Dis..

[23] Gupta K., Hooton T.M., Naber K.G., Wullt B., Colgan R., Miller L.G., Moran G.J., Nicolle L.E., Raz R., Schaeffer A.J. (2011). Infectious diseases society of America; European society for microbiology and infectious diseases. International clinical practice guidelines for the treatment of acute uncomplicated cystitis and pyelonephritis in women: a 2010 update by the Infectious Diseases Society of America and the European Society for Microbiology and Infectious Diseases. Clin. Infect. Dis..

[24] Haggard E., Hagedorn M., Bookstaver P.B., Justo J.A., Kohn J., Al-Hasan M.N. (2018). Minimum acceptable susceptibility of empirical antibiotic regimens for gram-negative bloodstream infections: a survey of clinical pharmacists. Infect. Dis. Clin. Practice.

[25] Schultsz C., Geerlings S. (2012). Plasmid-mediated resistance in *Enterobacteriaceae* changing landscape and implications for therapy. Drugs.

[26] Lambert P.A. (2002). Mechanisms of antibiotic resistance in *Pseudomonas aeruginosa*. J R Soc Med.

[27] Potron A., Poirel L., Nordmann P. (2015). Emerging broad-spectrum resistance in *Pseudomonas aeruginosa* and *Acinetobacter baumannii*: Mechanisms and epidemiology. Int. J. Antimicrob. Agents.

[28] Kiser T.H., Obritsch M.D., Jung R., MacLaren R., Fish D.N. (2010). Efflux pump contribution to multidrug resistance in clinical isolates of *Pseudomonas aeruginosa*. Pharmacotherapy.

[29] Liang B., Wheeler J.S., Blanchette L.M. (2016). Impact of combination antibiogram and related education on inpatient fluoroquinolone prescribing patterns for patients with health care-associated pneumonia. Ann. Pharmacother..

[30] Hammer K.L., Justo J.A., Bookstaver P.B., Kohn J., Albrecht H., Al-Hasan M.N. (2017). Differential effect of prior beta-lactams and fluoroquinolones on risk of bloodstream infections secondary to *Pseudomonas aeruginosa*. Diagn. Microbiol. Infect. Dis..

[31] Cha M.K., Kang C.I., Kim S.H., Cho S.Y., Ha Y.E., Wi Y.M., Chung D.R., Peck K.R., Song J.H., Korean Network for Study on Infectious Diseases (KONSID) (2015). In vitro activities of 21 antimicrobial agents alone and in combination with aminoglycosides or fluoroquinolones against extended-spectrum-β-lactamase-producing *Escherichia coli* isolates causing bacteremia. Antimicrob. Agents Chemother..

[32] Sader H.S., Castanheira M., Flamm R.K., Farrell D.J., Jones R.N. (2014). Antimicrobial activity of ceftazidime-avibactam against Gram-negative organisms collected from U.S. medical centers in 2012. Antimicrob. Agents Chemother..

[33] Farrell D.J., Flamm R.K., Sader H.S., Jones R.N. (2013). Antimicrobial activity of ceftolozane-tazobactam tested against *Enterobacteriaceae* and *Pseudomonas aeruginosa* with various resistance patterns isolated in U.S. hospitals (2011-2012). Antimicrob. Agents Chemother..

